# Calcium affects on vascular endpoints

**DOI:** 10.1186/1743-7075-9-24

**Published:** 2012-03-27

**Authors:** Vaishali B Patel, James L Vacek, Leland Graves, Rajib K Bhattacharya

**Affiliations:** 1 Division of Endocrinology, Metabolism & Genetics, The University of Kansas Medical Center, 3901 Rainbow Blvd, Mailstop 2024, Kansas City, KS 66160, USA; 2 Mid America Cardiology, The University of Kansas Medical Center, 3901 Rainbow Blvd, Mailstop 4023, Kansas City, KS 66160, USA

## Abstract

Calcium is one of the most abundant minerals in the body and its metabolism is one of the basic biologic processes in humans. Although historically linked primarily to bone structural development and maintenance, calcium is now recognized as a key component of many physiologic pathways necessary for optimum health including cardiovascular, neurological, endocrine, renal, and gastrointestinal systems. A recent meta-analysis published in August 2011 showed a potential increase in cardiovascular events related to calcium supplementation. The possible mechanism of action of this correlation has not been well elucidated. This topic has generated intense interest due to the widespread use of calcium supplements, particularly among the middle aged and elderly who are at the most risk from cardiac events. Prior studies did not control for potential confounding factors such as the use of statins, aspirin or other medications. These controversial results warrant additional well-designed studies to investigate the relationship between calcium supplementation and cardiovascular outcomes. The purpose of this review is to highlight the current literature in regards to calcium supplementation and cardiovascular health; and to identify areas of future research.

## Introduction

Calcium is one of the most abundant minerals in the body and its metabolism is one of the basic biologic processes in humans. Although historically linked primarily to bone structural development and maintenance, it is now recognized as a key aspect of many physiologic pathways necessary for optimum health including the cardiovascular, neurological, hormonal, renal, and gastrointestinal systems. Calcium serves as a cofactor for many extracellular enzymes, most notably the enzymes of the coagulation cascade, and as a source of calcium ions that function as signaling molecules for a great diversity of intracellular processes. These processes include automaticity of nerve and muscle; contraction of cardiac, skeletal, and smooth muscle; neurotransmitter release; and various forms of endocrine and exocrine secretion. Our review will describe the biology and basic physiology of calcium metabolism in humans, the present status of recommendations for intake and supplementation, the traditional role of calcium for optimum maintenance of the skeletal system, and then discuss in detail the relevance of calcium in cardiovascular health as well as several cardiac and vascular disease states.

### Current status of knowledge

The body of the average adult contains about 1000 gram of calcium of which 99% is located in the mineral phase of bone as hydroxyapatite crystals [Ca_10 _(PO_4_)_6_(OH)_2_]. These crystals play a key role in the mechanical weight-bearing properties of bone, serves as a source of calcium to support a number of calcium-dependent biological systems and to maintain blood ionized calcium within normal range. The remaining 1% of total body calcium is located in the blood, extracellular fluid and soft tissues. Of the total calcium in blood, the ionized fraction (45%) is the biologically functional portion and can be measured clinically. Most clinical laboratories report total serum concentrations. Forty-five percent of the total calcium in blood is bound to plasma, proteins notably albumin and up to 10% is bound to anions such as phosphate and citrate. Concentrations of total calcium in normal serum generally range between 8.5 and 10.5 mg/dl (2.12-2.62 mM).

#### Mineral homeostasis

**Figure 1:**
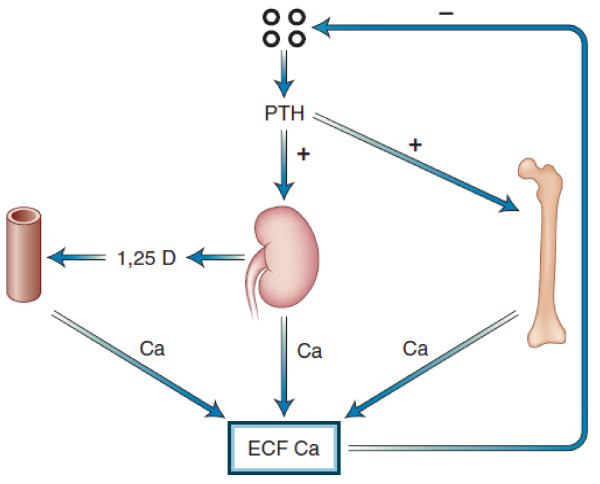
**Parathyroid hormone (PTH)-calcium feedback loop that controls calcium homeostasis**. Four organs-the parathyroid glands, intestine, kidney, and bone-together determine the parameters of calcium homeostasis. +, positive effect; -, negative effect; 1,25 D, 1,25-dihydroxyvitamin D3; ECF, extracellular fluid.

The skeleton, gut and the kidney play a major role in assuring calcium homeostasis. Overall in a typical individual, if 1000 mg of calcium is ingested per day, about 200 mg will be absorbed [1]. Approximately 10 gram of calcium will be filtered daily through the kidney and most will be reabsorbed, with about 200 mg being excreted in the urine [1]. The skeleton, a storage site of approximately 1 kg of calcium, is the major calcium reservoir in the body. Ordinarily as a result of normal bone turnover, approximately 500 mg of calcium is released from bone per day, and the equivalent amount is deposited per day [1]. Parathyroid hormone (PTH) enhances bone resorption and liberates both calcium and phosphate from the skeleton. PTH also enhances calcium re-absorption in the kidney while at the same time inhibiting phosphate re-absorption producing phosphaturia. Hypocalcemia and PTH itself can both stimulate the conversion of the inert metabolite of Vitamin D, 25-OH Vitamin D3 to the active moiety 1, 25-dihydroxy Vitamin D3 which in turn enhances intestinal calcium absorption (see Figure 1).

## Background

### Dietary calcium

Adequate calcium and Vitamin D intake along with regular exercise is an important aspect of bone health. The body cannot produce calcium, which must be ingested orally either through foods naturally rich in calcium, calcium fortified foods, or calcium supplements. Dairy products are naturally high in calcium. Milk, yogurt, and cheese are rich natural sources of calcium and are the major food sources of calcium in the United States [2]. Calcium-fortified foods and calcium supplements are helpful for people who are unable to get enough calcium in their regular diets.

#### IOM recommendations on calcium intake

Intake recommendations for calcium and other nutrients are provided in the Dietary Reference Intakes (DRIs) developed by the Food and Nutrition Board (FNB) at the Institute of Medicine of the National Academies (see table 1) [2].

**Table 1:** Dietary Reference Intakes of calcium

Life Stage Group	Estimated Average Requirements (mg/day)	Recommended Dietary Allowance (mg/day)	Upper Level Intake (mg/day)
Infants 0-6 months	200	200	1000
Infants 6-12months	260	260	1500
1-3 years	500	700	2500
4-8 years	800	1000	3000
9-13 years	1100	1300	4000
14-18 years	1100	1300	4000
19-30 years	800	1000	4000
31-50 years	800	1000	4000
51-70 years	800	1000	4000
51-70 year old female	1000	1200	4000
71+ year old	1000	1200	4000
14-18 year old pregnant/lactating	1100	1300	4000
19-50 year old pregnant//lactating	800	1000	4000

### Calcium intake and status

The Institute of Medicine defines the Adequate Intake (AI) of calcium as 1,000 mg/day for individuals aged 19 to 50 years and 1,200 mg/day for persons older than age 51 years, with the tolerable upper intake level (UL) of 2,500 mg/day. However, results from the National Health and Nutrition Examination Survey (NHANES) found that only 15% of men and eight percent of women older than 71 years had dietary calcium intakes meeting AI levels [3]. Mean calcium intake levels for males ranged from 871 to 1,266 mg/day depending on life stage group; for females the range was 748 to 968 mg/day [3]. Overall, females are less likely than males to get recommended intakes of calcium from food. About 43% of the U.S. population (almost 70% of older women) uses dietary supplements containing calcium [3]. Overall 60 percent of women over 60 report taking a dietary supplement containing calcium. About 30% of the calcium available in foods gets absorbed. Absorption varies depending upon the type of food consumed [2]. The efficiency of absorption decreases as calcium intake increases [2]. Absorption decreases to 15%-20% in adulthood (though it is increased during pregnancy) and continues to decrease as people age; therefore, recommended calcium intakes are higher for females older than 50 years and for both males and females older than 70 years [2,4,5]. When calcium intake is low or ingested calcium is poorly absorbed, bone breakdown occurs as the body uses its stored calcium to maintain normal biological functions

### Calcium supplements

Surveys have shown that many Americans are taking calcium supplements. The two main forms of calcium in these supplements are carbonate and citrate. Calcium supplements contain varying amounts of elemental calcium. For example, calcium carbonate is 40% calcium by weight, whereas calcium citrate is 21% calcium.

The amount of calcium absorbed from supplements depend on the total amount of elemental calcium consumed at one time; as the amount increases, the percentage absorption decreases. Absorption is highest in doses ≤500 mg of elemental calcium at one time [2]. Supplementing calcium in a divided dose regimen can, for the same ingested total load as in a single dose regimen, deliver as much as 80%-100% more calcium into the body [6]. Absorption of calcium from carbonate requires an acidic environment and so calcium carbonate should be taken with meals. Individuals taking medications that decrease stomach acid like proton pump inhibitors or H2-blockers should use calcium citrate supplements. Calcium citrate should be used in individuals with suspected achlorhydria, inflammatory bowel disease, or absorption disorders; however calcium carbonate is absorbed normally if taken with food, even in the presence of these conditions [7].

### Calcium and health

#### Calcium and bone health

Bone mass increases during periods of growth in childhood and adolescence, reaching a peak around age 30 years. Adequate intake of calcium and Vitamin D is essential throughout childhood, adolescence and early adulthood. In pre-pubertal children whose average dietary intake of calcium approximated the recommended dietary allowance, calcium supplementation increased the rate of increase in bone mineral density [8]. If the gain persists, peak bone density should be increased thereby reducing risk of fracture.

Bone undergoes continuous remodeling, with constant resorption and deposition of calcium into new bone. In aging adults, particularly among postmenopausal women, bone breakdown exceeds formation, resulting in bone loss that increases the risk of osteoporosis over time [2]. Calcium supplements mitigate the aging-related bone loss. They also preserve bone in premenopausal women. A controlled 2 year longitudinal trial of 248 perimenopausal women observed that a daily supplementation with 1000 or 2000 mg of calcium significantly reduced bone turnover and bone loss from the lumbar spine in premenopausal and early menopausal women for the two years of study and an additional year of follow up [9]. The responsiveness of postmenopausal women to supplementation with calcium appears to depend on their menopausal age. In women who are within the first 5 years of menopause, bone loss is attenuated but not arrested by added calcium. The maximal effect appears to occur with supplement dosages of approximately 1000 mg elemental calcium per day [9].

#### Calcium supplementation and cardiac health

Calcium supplements are widely used in the United States and other developed nations, traditionally to avoid osteopenia and its complications, but now often given along with vitamin D supplementation for maintenance of optimum health for other physiologic processes, including the cardiovascular system Many prior studies have shown varying results in regards to calcium supplementation and cardiovascular disease risk and outcomes.

##### Randomized placebo controlled trials

A secondary analysis of a randomized placebo controlled trial of calcium supplements versus placebo in post-menopausal women for 5 years showed a statistically significant increase in MI [10]. The composite end point of myocardial infarction (MI), stroke or cerebrovascular accident (CVA), or sudden death was higher in the calcium supplemented group (*P *= 0.008), relative risk was 1.66 [10]. When the events were adjudicated, statistical significance was no longer found. The baseline characteristics of the participants in this study were not adjusted for cardiovascular risk nor was there adequate documentation for concomitant cardiovascular medications.

The Women's Health Initiative trial, another randomized controlled study did not show any significant cardiovascular risk from calcium supplements. This was a RCT of calcium 500 mg plus Vitamin D 200 IU twice daily versus placebo in 36,282 post-menopausal women 50 to 79 years at 40 clinical sites, where CVD was a pre-specified secondary efficacy outcome. This study revealed that calcium/vitamin D supplementation neither increased nor decreased the risk for CHD or stroke in generally healthy postmenopausal women throughout the 7-year duration of this randomized trial [11]. Alimitation of this trial was poor adherence to the supplements. At the end of the trial (mean follow-up, 7 years), 76% of participants were taking "some" study pills, and 59% were taking-80% of their study medication. This may have played a role in neutral effects on the cardiovascular risk. Another limitation of the trial was that women in the placebo group were allowed to continue their own calcium supplements. About 54% of participants were taking personal calcium supplements at randomization, which could have masked the difference between the 2 groups on the effect of calcium and vitamin D on the risk of cardiovascular events. In addition a high percentage of women were using hormone replacement therapy, which may have an independent role in cardiovascular mortality.

A 5-year randomized, controlled trial (RCT) and 4.5 years of post-trial follow-up showed that 1200 mg of calcium (as calcium carbonate) daily does not significantly increase the risk of atherosclerotic vascular disease in elderly women [12]. Further analysis of this RCT suggested that calcium supplementation may reduce the risk of hospitalization and mortality in patients with preexisting atherosclerotic cardiovascular disease [12]. In addition, the incidence of the heart failure outcome was reduced in calcium-treated patients at 9.5 years [12]. The strengths of this study over previous studies include the complete follow-up of all 1460 participants and that the adverse events were analyzed to provide clear end points [12].

## Meta-analysis

A meta-analysis of 11 studies which included 12000 participants showed that allocation to calcium supplements (> 500 mg/day for more than a year) was associated with increased risk of MI and that calcium supplementation was associated with 30% increase in incidence of MI, and smaller non-significant increase in risk of CVA and death [13]. None of the trials had CV events as the primary end points and data on CV events were not gathered in a standardized manner. Incomplete or no data on CV outcomes were available in 7 trials comprising about 15% of participants. None of the trials were designed to have the baseline characteristics of the subjects equalized in terms of cardiac risk factors. The trials did not have information about the use of statins, ACE inhibitor therapy or previous history of coronary artery disease. The studies which compared calcium plus Vitamin D with placebo were excluded, so the results may not apply to co-administered calcium and Vitamin D supplements. Many commonly used calcium supplements consumed or available currently in the United States have Vitamin D included.

### Prospective cohort study

A prospective cohort study of 34,486 postmenopausal Iowa women 55-69 years old, without a history of ischemic heart disease was analyzed to investigate whether greater intakes of calcium, vitamin D, or milk products may protect against ischemic heart disease mortality. Eight year follow up suggested that, among postmenopausal women, the risk of dying of ischemic heart disease may be reduced by consuming relatively high levels of calcium [14]. There was an estimated statistically significant 33 percent reduction in risk for persons in the highest quartile of total calcium intake (i.e., high whether due to diet, supplements, or both). Limitation of the study was that the duration of supplemental vitamin and mineral use was not known.

#### Observational cohort study

An observational cohort study of 9910 women aged 60-89 years with no pre-existing heart disease or stroke history showed that after 2 years of calcium and vitamin D supplementation there was no increase in cardiovascular events or deaths compared to women who received minimal supplementation [15]. How do calcium supplements increase the risk of vascular events.

The mechanisms by which calcium supplements might increase the risk of myocardial infarction are unclear and speculative. A possible explanation is that calcium supplements acutely increase serum calcium levels to a modest degree stimulating the calcium sensing receptors on the cells of the vessel wall and have deleterious effects [16]. It is speculated that it may also increase vascular calcification and thereby cardiovascular events [13]. Other possible mechanisms may be increased coagulability, as the time to initiation of coagulation is inversely related to ionized calcium concentration [16]. Increased arterial stiffness may play a role in vascular events [16].

## Calcium supplementation and arterial calcification

The effect of calcium supplementation on arterial calcification is unclear. Post-trial coronary artery calcification (CAC) measurements using cardiac CT were similar in women randomized to calcium/vitamin D supplementation (calcium/D) and those receiving placebo. Treatment with moderate doses of calcium plus vitamin D3 did not appear to alter coronary artery calcified plaque burden among postmenopausal women [17].

In subjects new to hemodialysis, baseline CAC (coronary artery calcification) score is a significant predictor of all-cause mortality. Use of calcium-containing phosphate binders in patients new to hemodialysis is associated with a rapidly progressive increase in the extent of CAC with resultant higher mortality [18]. Total body calcium score and progression of CAC score in predialysis patients was shown to be higher with the use of calcium containing phosphate binders versus non calcium containing phosphate binders [19]. Calcium supplementation can hypothetically increase arterial calcification and cardiovascular events, so calcium supplements should be used with caution in patients with renal disease. People with impaired renal function who take calcium supplements may be at higher risk of cardiovascular problems [20].

### Calcium supplementation and blood pressure

Relationship between calcium intake/supplementation and blood pressure or risk of hypertension is unclear. The Women's Health Initiative study revealed that over a median follow-up time of 7 years, there was no significant difference in the mean change over time in systolic blood pressure (0.22 mm Hg, 95% CI -0.05 - 0.49 mm Hg) and diastolic blood pressure (0.11 mm Hg, 95% CI -0.04 - 0.27 mm Hg) between the active and placebo treatment groups [11]. In postmenopausal women, calcium plus vitamin D3 supplementation did not reduce either blood pressure or the risk of developing hypertension over seven years of follow up.

A prospective cohort study of 28,886 US women aged 45 years, revealed that intakes of low-fat dairy products, calcium, and vitamin D were each inversely associated with risk of hypertension in middle-aged and older women, suggesting their potential roles in the primary prevention of hypertension and cardiovascular complications during 10 years of follow-up [21]. This study was limited to white women.

### Calcium and lipids

Limited data is available in regards to calcium intake/supplementation on lipid profile. RCT of supplementation of calcium citrate in normal older women showed beneficial changes in circulating lipids in post-menopausal women. After 12 months, HDL cholesterol levels and the HDL cholesterol to LDL cholesterol ratio had increased more in the calcium group than in the placebo group [21]. This was largely due to a 7% increase in HDL cholesterol levels in the calcium group, with a non-significant 6% decline in LDL cholesterol levels. There was no significant treatment effect on triglyceride level [22]. Calcium possibly binds to fatty acids and bile acids in gut resulting in poor fat absorption. Other possible mechanism include increased lipolysis as a result of inhibition of PTH and 1, 25 (OH)_2 _Vitamin D.

### Calcium and Diabetes

The available evidence on the role of calcium supplementation in the development of diabetes is limited, but a systematic review and meta-analysis suggests that combined vitamin D and calcium supplementation may have a role in the prevention of T2DM only in populations with glucose intolerance, who are at high risk of developing diabetes [23].

## Conclusion

Osteoporosis is a growing health problem and a common cause of morbidity. Adequate calcium intake is essential for maintaining good bone health. As discussed above, calcium supplements are commonly used in US to satisfy the daily dietary recommendations of calcium. Calcium supplements modestly increase bone density and have marginal efficacy against fracture. The number of patients needed to treat with calcium and or Vitamin D supplementation for 5 years to prevent one fracture is estimated to be 48 [24].

Cardiovascular disease is the main cause of mortality and morbidity in US. Recently concern has been raised regarding an increased risk of MI associated with calcium supplements [10], raising questions about the safety of calcium supplements with respect to cardiovascular disease risk. Based on reported data, if 1000 people were given calcium supplements for 5 years, theoretically there would be an additional 14 myocardial infarction [10]. In the Iowa Women's Health Study, higher calcium intake was associated with reduced ischemic heart disease mortality in postmenopausal women [14]. Another RCT revealed that calcium supplementation does not significantly increase the risk of cardiovascular events [12].

These varying results should prompt more well designed studies to investigate the relationship between calcium supplements and cardiovascular health. Very few studies in the past have been controlled for factors such as the use of statins, aspirin, ACE inhibitors, ARBs or other medications and further research is needed in these matters In addition some of the major studies have not adjudicated CV events as a major outcome leading to potential data collection bias. Adequate calcium intake/supplementation is essential in childhood, adolescence and adulthood for optimal bone health. Increased cardiovascular risk associated with calcium supplementation in young women would translate to a large burden of disease. This is speculation and warrants further research. Osteoporosis is becoming an increasing public problem in males as well. Paucity of data exists on relationship of calcium supplementation and cardiovascular risk in males. Further research is needed to clarify the magnitude of this problem and identify the factors associated with risk and mechanism of adverse effects of calcium supplementation on cardiovascular disease in males and females.

## Competing interests

Vaishali B. Patel, James Vacek, and Leland Graves III declare that they have no competing interests.

Rajib K. Bhattacharya: Speaker for Amgen: Bristol-Myers Squibb, Novartis: Solvay and Sanofi Aventis Auxilium: advisor/consultant, steering committee.

## Authors' contributions

VP and RB did the literature search and drafted the manuscript, JV, RB and LG revised it and provided other necessary literature for drafting the manuscript. All authors read and approved the final manuscript.
